# Mechanisms of TIGIT-driven immune suppression in cancer

**DOI:** 10.1186/2051-1426-2-S3-O13

**Published:** 2014-11-06

**Authors:** Sema Kurtulus, Kaori Sakuishi, Huiyuan Zhang, Nicole Joller, Dewar Tan, Mark Smyth, Vijay Kuchroo, Ana Anderson

**Affiliations:** 1 Center for Neurologic Diseases Brigham & Women's Hospital, Boston, MA, USA; 2 The University of Tokyo, Tokyo, Japan; 3 Peter MacCallum Cancer Centre, Queensland Institute of Medical Research, Queensland, Australia

## 

TIGIT is a co-inhibitory molecule that limits T cell proliferation and activation. TIGIT expression has been recently shown to identify a subset of regulatory T cells (Treg) that specifically suppresses Th1 and Th17 responses; however its role in tumor immunity has not been examined. Here, we determined whether TIGIT has a role in the suppression of anti-tumor immune responses. We found that TIGIT is highly up-regulated on Treg and CD8+ tumor-infiltrating lymphocytes (TILs) in multiple pre-clinical cancer models. Importantly, TIGIT expression is strongly associated with expression of other co-inhibitory molecules; PD-1, Tim-3 and Lag-3 and with production of IL-10 in Treg and CD8+ TILs. Moreover, TIGIT+ CD8+ TILs display an exhausted phenotype determined by decreased production of IL-2 and TNF-α. To understand whether TIGIT acts as a checkpoint in anti-tumor response, we monitored growth of implanted B16 melanoma in TIGIT^-/- ^mice and found that absence of TIGIT significantly delayed tumor growth. As TIGIT is expressed on both T cells (Treg and CD8+) and NK cells in cancer, we addressed the role of TIGIT in these subsets in driving immune suppression. Our data indicate that TIGIT may play a dominant role in Treg in that deficiency of TIGIT in Treg alone results in better control of tumor growth and a heightened proliferative response in the draining lymph nodes and spleens of tumor-bearing mice. Finally, we show that a TIGIT blocking antibody can be used therapeutically to decrease tumor growth and that blockade of TIGIT synergizes with Tim-3 blockade to maximally decrease tumor growth. Our study is the first report showing that TIGIT acts as an immune checkpoint in cancer. Importantly, our data indicate that TIGIT and Tim-3 synergize to suppress anti-tumor responses and targeting these two molecules could provide a therapeutic effect on tumor growth.

